# Epidemiology and Molecular Characterizations of Coronavirus from Companion Animals Living in Chengdu, Southwest China

**DOI:** 10.1155/2023/5056492

**Published:** 2023-03-30

**Authors:** Yingying Zhang, Lan You, Yue Zou, Xun He, Shanshan Wu, Fen Yang, Xin Xu, Xiaofang Pei, Jiayi Chen

**Affiliations:** ^1^West China School of Public Health and West China Fourth Hospital, Sichuan University, 16#, Section 3, Renmin Road South, Chengdu, 610041, Sichuan, China; ^2^West China Tianfu Hospital, Sichuan University, 3966#, Section 2, South Second Section, Tianfu Avenue, Chengdu 610200, Sichuan, China; ^3^Chengdu Center for Disease Control and Prevention, 4#, Longxiang Road, Chengdu 610041, Sichuan, China

## Abstract

The recent COVID-19 pandemic has once again caught the attention of people on the probable zoonotic transmission from animals to humans, but the role of companion animals in the coronavirus (CoV) epidemiology still remains unknown. The present study was aimed to investigate epidemiology and molecular characterizations of CoVs from companion animals in Chengdu city, Southwest China. 523 clinical samples from 393 animals were collected from one veterinary hospital between 2020 and 2021, and the presence of CoVs was detected by end-point PCR using pan-CoV assay targeting the RdRp gene. Partial and complete *S* genes were sequenced for further genotyping and genetic diversity analysis. A total of 162 (31.0%, 162/523) samples and 146 (37.2%, 146/393) animals were tested positive for CoVs. The positive rate in rectal swabs was higher than that in eye/nose/mouth swabs and ascitic fluid but was not statistically different between clinically healthy and diseased ones. Genotyping identified twenty-two feline enteric coronavirus (FCoV) I, four canine enteric coronavirus (CECoV) I, fourteen CECoV IIa, and one CECoV IIb, respectively. Eight complete *S* genes, including one canine respiratory coronavirus (CRCoV) strain, were successfully obtained. FCoV strains (F21071412 and F21061627) were more closely related to CECoV strains than CRCoV, and C21041821-2 showed potential recombination event. In addition, furin cleavage site between S1 and S2 was identified in two strains. The study supplemented epidemiological information and natural gene pool of CoVs from companion animals. Further understanding of other functional units of CoVs is needed, so as to contribute to the prevention and control of emerging infectious diseases.

## 1. Introduction

The emergence, pandemicity, and devastating impact of SARS-CoV-1, MERS-CoV, and SARS-CoV-2 for the past two decades have emphasized the zoonotic origin of coronaviruses and their potential tremendous threat posed on public health [1–3]. Queries about the appearance of SARS-CoV-2 and transmission chains into humankind remain to be answered. Susceptible animals may not only act as intermediate hosts posing disease risks but also may prompt the emergence of host-range-expanding and species-barriers-crossing strains [4–6]. Therefore, extensive attentions have been drawn to coronavirus from animals, especially those companion and agricultural animal species that have close contact with humans.

Cats and dogs are very common companion animals living in vicinity with humans. About 50% of families raise companion animals in Europe and the USA [7], and there were 62.94 million people raising more than 100 million pet dogs and cats in China in 2020 [8]. With the widespread of SARS-CoV-2, a number of companion animals were found positive for COVID-19 infections, where pet owners and zoo employees might have facilitated the transmissions [9–11]. Further experimental studies have confirmed that cats are highly susceptible to SARS-CoV-2 infections and can transmit to other cats, causing asymptomatic or mild respiratory signs, indicating that pets can be the potential intermediate hosts that are likely neglected [12–14].

Companion animals are natural reservoirs of many CoVs, including feline enteric coronavirus (FCoV), feline infectious peritonitis virus (FIPV), canine enteric coronavirus (CCoV), and canine respiratory coronavirus (CRCoV), which all are responsible for causing enteric infection to lethal immuno-inflammatory systemic disease [15]. Complex and variable recombination of *S* gene occurs continuously between these CoVs. For example, double homologous recombination of type I FCoV and CCoV gave rise to the emergence of type II FCoV [16]. Notably, recent evidence reported that canine-feline-porcine-like Alpha CoVs have been detected in humans in different countries and can be connected with human respiratory illness [17, 18]. Abovementioned existing data once again highlight the potential threat of zoonotic CoVs on public health and the importance to conduct surveillance for them.

Thus, in this study, a continual epidemiological research was conducted to investigate the prevalence, clinical features of CoVs infections in companion animals in Southwest China. Partial and complete *S* genes were used to analyze the genetic diversity and possible recombinations of the CoV strains identified. A better understanding of CoVs from companion animals, prevalence, genetic information, and possible cross-species transmission, may contribute to public management practices for emerging zoonotic CoVs strains.

## 2. Materials and Methods

### 2.1. Specimens' Sourcing

The study was performed in accordance with the OIE Terrestrial animal health code, which was prospectively conducted between July 2020 and December 2021 in one veterinary hospital in Chengdu, Southwest China. Sample collections and data collection were approved by the veterinary hospital and the pets' owners. Eye/nose/mouth swabs, rectal swabs, and ascitic fluid samples were collected by trained veterinarians using viral transport media (Qingdao Hope Bio-Technology Co. Ltd., China) with a volume of 3 ml. For all samples, the companion animals' age, breed, gender, health condition (healthy or sick), and collection date were recorded. All samples were shipped on dry ice and were stored at −80°C freezer until further analysis.

### 2.2. Detection of Coronavirus

The samples were centrifuged at 3000 r/min for 10 min, and viral RNA was extracted from 200 *μ*l of the supernatant according to the protocol (Virus DNA/RNA Extraction Kit II, Geneaid Biotech). The extracted RNA was reverse transcribed into cDNA using iScript™ cDNA Synthesis Kit (Bio-Rad, California, US) and then stored at −20°C until required. Coronavirus infection was detected by end-point PCR targeting the RNA-dependent RNA polymerase (RdRp) gene using pan-CoV assay as previously described [19] (Table S1).

### 2.3. Genotyping Based on Partial *S* Genes

In order to differentiate the FCoV (type I and type II) and CCoV (type I, IIa and IIb) strains, partial region of the *S* gene was amplified according to the protocol [20–22] (Table S1). The PCR products consistent with the expected size were determined by sequencing (Tsingke Biotechnology, Beijing, China).

### 2.4. Sequence Analysis Based on Complete *S* Genes

The full-length of *S* genes from the positive samples was amplified according to the designed primers (Tables S2 and S3). The complete *S* sequences were assembled using SnapGene software (version 6.0). Multiple sequence alignments and the homology analysis of the nucleotide (nt) and deduced amino acid (aa) sequence were performed using the MEGA 11.0 software (freely available at https://www.megasoftware.net/). All data on protein hydrophobicity, stability, and amino acids at S1/S2 cleavage sites were obtained from ProtParam. All the obtained sequences from this study have been deposited in the GenBank database under the following accession numbers: OQ407503∼OQ407523, OQ442209, and OQ351912∼OQ351919.

### 2.5. Phylogenetic and Recombination Analysis

The phylogenetic tree was constructed by MEGA 11.0 software using the maximum likelihood method, with 1000 bootstrap replicates. The reference *S* genes of typical FCoV, CCoV, and CRCoV strains from China and other districts were retrieved from the National Center for Biotechnology Information (NCBI) nucleotide database. Analysis of potential recombination events was conducted using RDP 4.0, and similarity plots and bootscanning analysis were carried out using the SimPlot software version 3.5.1.

### 2.6. Statistical Analysis

A positive case was considered if any of the samples from each animal was tested positive for CoV. The differences of CoV prevalence between different clinical samples, as well as the correlation of CoV infection with clinical signs, gender, age, and season were carried out by Chi-square analysis or Fisher's exact test in IBM SPSS Statistics. The threshold for statistical significance was set at *p* value <0.05.

## 3. Results

### 3.1. Total Prevalence of CoV

A total of 523 clinical samples (180 eye/nose/mouth swabs, 336 rectal swabs, and 7 ascitic fluid) from 393 companion animals (123 dogs and 270 cats) were obtained from veterinary hospitals in Chengdu, Southwest China. Of the 523 samples, 31.0% (162/523) were tested positive for CoV, among which there were 21.1% (38/180), 28.6% (2/7), and 36.3% (122/336) for eye/nose/mouth swabs, ascitic fluid, and rectal swabs, respectively, with rectal swabs having the highest detection rate. A total of 37.2% (146/393) of companion animals were positive for CoV, and more than half (58.5%, 58/126) of the animals from whom both swabs were collected were tested positive for CoV.

The mean age was 1.0 years (1 month to 8 years) for cats and 1.9 years (1 month to 20 years) for dogs. Young animals (<6 M) tended to have a higher proportion of CoV infection than the older ones (>6 M) (*p* < 0.05). The seasonal detection rates ranged from 16.3% to 48.8%, and CoV infections were more likely to occur in winter months (December to February) and spring months (March to May). However, the differences of CoV prevalence were not statistically significant in different gender and health status (Table 1). Diarrhea and emesis are the main clinical signs for FCoV and CCoV infection (Table S4). The distribution of coronavirus groups, genotypes, and recombination event between symptomatic and asymptomatic animals was also analyzed and is displayed in Table 2.

### 3.2. Genotyping and Sequence Analysis of the CoV Strains

**Figure 1:**
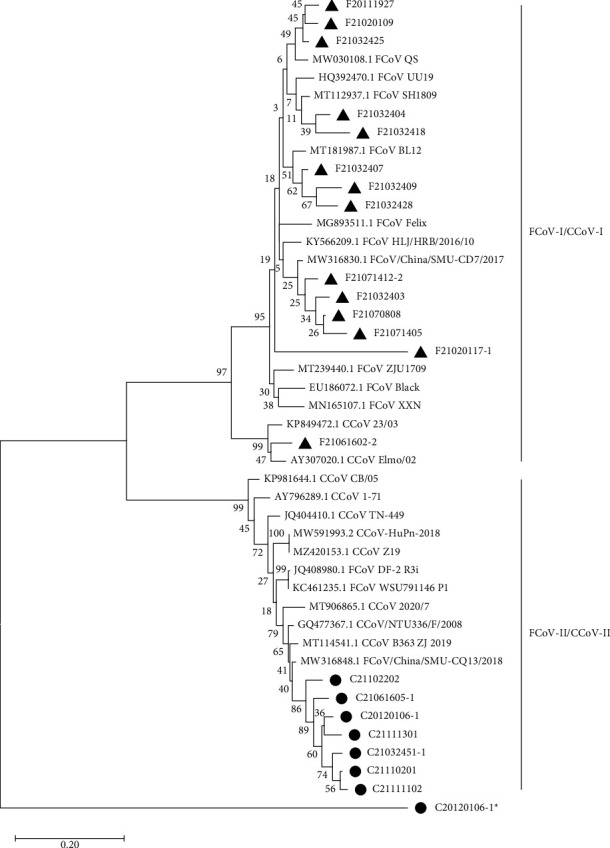
Phylogenetic analysis of partial spike (S) gene sequences. FCoV strains from this study are indicated by black triangle and CCoV strains from this study are indicated by black circle. C20120106-1 (CCoV type IIa) and C20120106-1^*∗*^ (CCoV type IIb) were isolated from the same sample. Numbers at the branches represent bootstrap values obtained in the phylogenetic analysis.

Forty partial *S* genes were successfully sequenced and indicated that 22 were positive for type I FCoV, 4 samples were positive for type I CCoV, and 14 for type II CCoV. The nucleotide homology of identified FCoV strains was 74.83%∼97.14% while the nucleotide homology between CCoV-II strains was 56.97%∼99.55%. Notably, nucleotide homology of CCoV IIb strain (C20120106-1) was ≤58.18% compared to other strains and showed nucleotide homology of 95.29% with reference strains CFBCoV-DM95/2003 (EF192159.1). The phylogenetic tree constructed was composed of two groups, namely, type II FCoV/type II CCoV and type I FCoV/type I CCoV (Figure 1). Fourteen strains belonged to the type II CCoV group, exhibiting close relationships with reference strains from Zhejiang (China) and British. A strain of FCoV-I (F21061602) was found to be more closely related to CCoV. Furthermore, type IIa and type IIb was identified in the same sample from an asymptomatic animal, and type IIb formed a single clade.

### 3.3. Coronavirus Spike Gene Characterization

In our study, the full-length of *S* genes from 8 strains was obtained. Two FCoV-I strains had a total length of 4404∼4407 bp, five CECoV (canine enteric coronavirus) strains with 4362 bp, and one CRCoV with 4092 bp, which encoded aa residues 1468∼1469, 1454, and 1364, respectively. The nucleotide and aa identities between the eight strains are displayed in Table S5. Moreover, CoVs were detected in both rectal swabs and eye/nose/mouth swabs from the same dog, which were identified as CECoV and CRCoV, respectively. The nucleotide and aa identities between the two strains were 52.99% and 27.57%.

**Figure 2:**
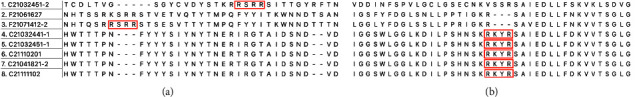
The cleavage sites of the amino acid sequences of the 8 strains in this study. (a) The cleavage site between S1 and S2. (b) The cleavage site in S2 subunit.

The aa sequences identities of the S1 and S2 subunit from the eight whole *S* genes and five CECoV whole *S* genes (Table S5) revealed the most significant variation in S1 subunit compared to the S2 subunit. By sequence alignment, two strains (FCoV F21071412-2 and CRCoV C21032451-2) contained protease cleavage motifs RSRR at position P792 and P765, respectively, whereas an obvious motif was not found in the other strain F21061627 (Figure 2). The five CECoV strains showed the presence of cleavage motif RKYR in S2 subunit.

### 3.4. Phylogenetic Analysis of *S* Gene

**Figure 3:**
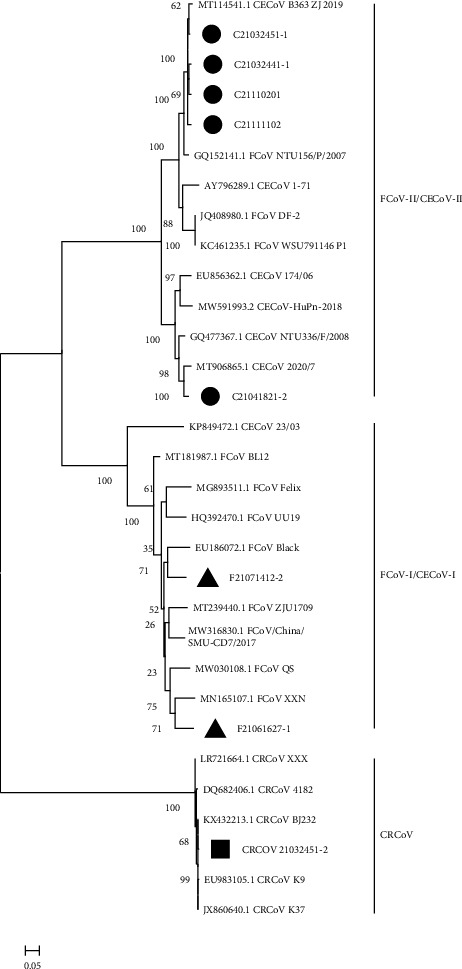
Phylogenetic tree of full-length spike (S) gene of CCoV, FCoV, and CRCoV. CCoV, FCoV, and CRCoV strains identified in this study are represented by black circle, black triangle, and black rectangle, respectively.

The phylogenetic tree generated based on the eight full-length *S* genes was composed of three groups, namely, type II FCoV/CECoV, type I FCoV/CECoV, and CRCoV. Four CCoV-II strains showed the closest relationship with CCoV B363 ZJ 2019 strain and FCoV NTU156/P/2007 strain that were previously identified in China, and one CCoV-II strain showed the closest relationship with another strain 2020/7 from Britain. In addition, the two FCoV-I strains from our samples were clustered together with the Australian strain Black and the Chinese strain XXN, respectively. Besides, one CRCoV strain identified in this study was closely related to the Chinese BJ232 strain and Korean K9 strain (Figure 3).

### 3.5. Recombination Analysis

**Figure 4:**
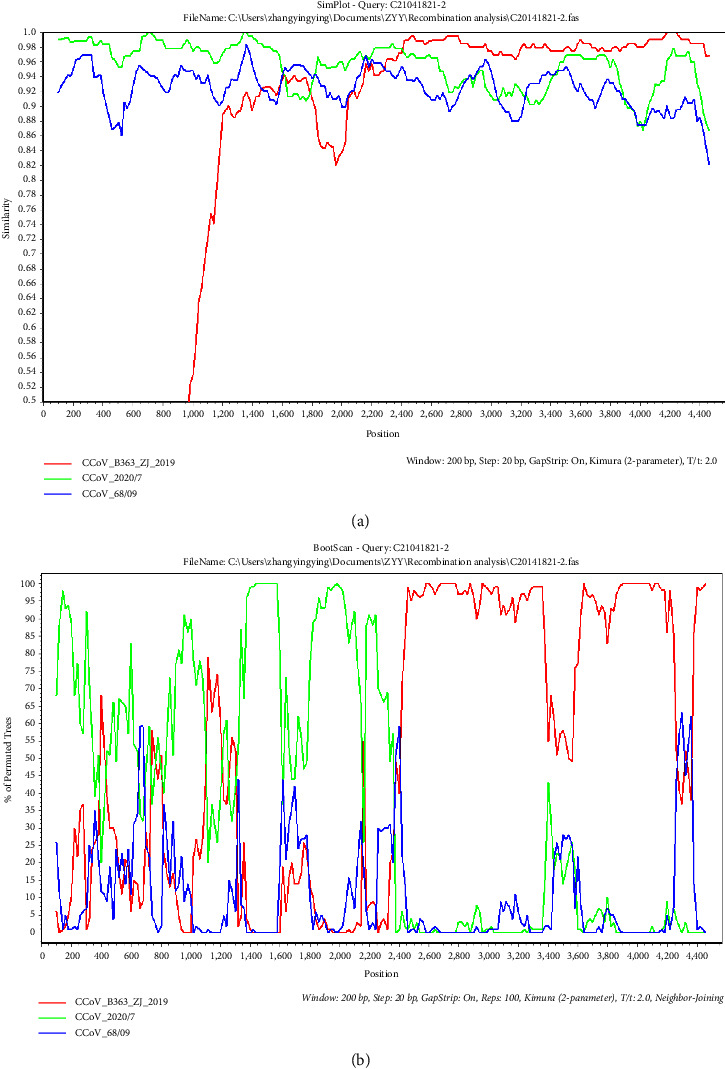
Genetic recombination analysis of the complete spike (S) of C21041821-2 strain. (a) The results of a SimPlot similarity analysis. (b) The results of a bootscanning analysis. The C21041821-2 strain was used as the query sequence and was compared with three other representative strains of CCoV-like viruses, including CCoV B363 ZJ 2019 (MT114541.1), CCoV 2020/7 (MT906865.1), and CCCoV 68/09 (HQ450377.1). A similarity of 1.0 indicates 100% identity with the nucleotide sequence. A window size of 100 bp, a step size of 20 bp, and 100 replicates were used as the default settings.

Recombination analysis indicated that strain C21041821-2 (identified in an asymptomatic animal) exhibited a strong recombination signal in seven methods, with a recombination score of 0.708. Similarity plots and bootscanning analyses were performed to confirm the recombination (Figure 4). C21041821-2 strain was used as a query sequence and was compared with CCoV B363 ZJ 2019 (MT114541.1), CCoV 2020/7 (MT906865.1), and CCoV 68/09 (HiQ450377.1). C21041821-2 possessed the highest similarity with the B363 ZJ 2019 strain between position 2400 and 4200. Moreover, the SimPlot analysis results revealed that strain C21041821-2 was related to B363 ZJ 2019, 2020/7, and 68/09, which further confirmed the occurrence of C21041821-2 strain genetic recombination.

## 4. Discussion

Since 1940, more than 60% of the emerging infectious diseases are zoonotic in nature [23], and cross-species transmission of CoVs tends to happen. Scientists have advocated One World One Health approach to intervene the transmission chain of diseases, and thus it is of great significance to identify the range of potential reservoirs, hosts, and susceptible animals.

The prevalence of CoVs in this study was relatively higher than that in Beijing, Heilongjiang, and overall prevalence in five provinces of China [24–26], but slightly lower than existing reports of diseased dogs in Japan and Greece [27, 28]. Factors such as districts, health status, multianimal environment, and gender may contribute to the different prevalence. The application of a newly developed molecular diagnostic method that is capable to detect most CoVs may partly explain the relatively high detection rate in this study. Nonspecific diagnostic tests such as pan-CoV assay can greatly increase detection rate and play an important role in CoV surveillance, but they may also lead to false positive results. Specific detection methods targeting certain CoVs are accurate but with low throughput and are more suitable for diagnosis. Therefore, it is better to choose suitable methods based on different scenarios, and developing high-throughput multiplex methods in the future is well recommended.

How to choose the efficient specimens in the surveillance setting is still worth considering. In previous epidemiological studies, samples are collected primarily based on clinical symptoms in these animals, such as fecal specimen for enteric disease, ascitic fluid for FIP, and nasopharyngeal swabs for respiratory disease. Eye/nose/mouth swabs and rectal swabs were both collected in our research, and the positive rate of CoVs in rectal swabs is higher than that in eye/nose/mouth swabs, suggesting that rectal swabs could be the preferred target specimen in surveillance settings. Besides, the combined detection rate from two types of samples is higher than that from single sample, indicating that eye/nose/mouth swabs could also be collected to improve detection rate.

Meanwhile, CoVs can also be detected in healthy animals other than diseased ones. The present study found a comparable prevalence of CoV carriage between asymptomatic and symptomatic animals and also identified mixed CCoV IIa/IIb infection and recombination event in asymptomatic animals, implying the important role of asymptomatic animal played in the spread of the disease. Asymptomatic infection may act as a silent vector for pathogens during disease outbreaks, posing a significant public health issue [29, 30]. Much is still unknown regarding the extent of occurrence and its role in transmission, and therefore, the impact of asymptomatic cases on the spread of CoV deserves further investigation.

According to the phylogenetic analysis of the partial *S* gene, the present study found a similar distribution of type I FCoV and type II CCoV compared to previous reports [24, 31–33], suggesting that the predominant endemic strain was type I FCoV and CCoV-IIa. There was also a mixed infection of two genotypes identified, which might accelerate the emergence of novel strains and increase tissue tropism. The recombination of *S* gene is a complex and unpredictable event that occurs frequently between different CoVs. For instance, the emergence of CCoV-IIb was a result of potential recombinant origin of type II CCoV and transmissible gastroenteritis virus (TGEV), a swine coronavirus, illustrating a possible interspecies transmission between dogs and pigs [21, 34]. Furthermore, the evolutionary results found that type I FCoV clusters with type I CCoV and type II FCoV clusters with CCoV type II, which indicate recombination events occurred between FCoV and CCoV on at least one of the hosts, suggesting that attention must be paid to monitor the cross-region transmission and recombination events in epidemic strains among different districts.

The viral entry of coronavirus primarily depends on recognition and binding of the *S* protein, which consisted of S1 and S2 subunit [35]. Notably, much attention has been paid on this specific sequence motif that mediates the hydrolysis of glycoproteins at the S1/S2 cleavage site and furin cleavage site. This site is crucial to facilitate infections for a variety of viruses, but yet, information about CoVs with furin cleavage sites is relatively scarce. Evidence obtained from human coronaviruses indicates that the existence of a furin cleavage site in *S* protein is essential for replication, pathogenesis, and transmission of SARS-CoV-2 [36, 37]. In this study, we identified cleavage motifs in one FCoV and one CRCoV strain. FCoV with a furin cleavage site was reported in 1998 in the United States and further were recovered in 2007 in the Netherlands [38]. CRCoV is a betacoronavirus and can cause mild to moderate upper respiratory disease [15]. CRCoV with a furin cleavage site was seldom reported. High sequence identity with bovine coronavirus (BCoV) and human coronavirus OC43 might partly explain the presence of this site [38, 39]. Recent publication focusing on the global CoVs with furin cleavage sites indicated that recombination frequency of this site may have been underestimated [38]. Whether the amino acid changes at furin sites could have the same impact on the pathogenicity of companion animals' CoVs remains to be discussed.

There are currently no licensed vaccines against FCoV in China, and eight-combination vaccine is available for CCoV prevention. CCoV vaccines are developed based on type IIa, but whether it protects against other variants is still uncertain [40]. Besides, the vaccine appears to protect dogs from disease but not from infection, which might partly explain asymptomatic carriage of CCoV [41]. Continuous surveillance of CoVs in companion animals can help better understand the circulation, evolution, and genetic diversity of these CoVs, so as to contribute to developing efficient and accurate diagnostics, therapeutics, and vaccines.

In conclusion, the current study described the epidemiological characteristics of CoVs in companion animals in Southwest China during 2020-2021. We reported comparable carriage rate of CoVs in both healthy and diseased animals, with the FCoV-I, CCoV-II being the predominant strain and two strains with furin cleavage sites identified. Partial RdRP gene of infectious bronchitis virus (IBV), a gamma coronavirus, was detected from both swabs of a severely diseased dog (C21041809), implying possible interspecies transmission from avians to mammals. Unfortunately, we are unable to confirm this potential event due to the sudden decease of this animal. Considering intimate relationship between pets and humans, potential cross-species transmissions of zoonotic CoVs (as well as other viruses) should be systemically monitored at the human-animal interface. In keeping with the One Health concept, a more thorough understanding of furin cleavage site and other functional units of CoVs is needed, so as to better develop therapeutic targets and universal vaccines and to prevent future epidemics and pandemics.

## Figures and Tables

**Table 1:** Correlation of the CoV prevalence with different clinical symptoms, gender, age and seasons.

Variables	Number of animals (*n* = 393)	CoV-positiveanimals (*n* = 146)	CoV-negativeanimals (*n* = 247)	*p* value
Clinical status				
Presenting clinical symptoms	170	67 (39.4%)	103 (60.6%)	>0.05
Healthy animals	223	79 (35.4%)	144 (64.6%)	
Gender				
Male	219	87 (39.7%)	132 (60.3%)	>0.05
Female	165	57 (34.5%)	108 (65.5%)	
Age				
≤6 months	181	78 (43.1%)	103 (56.9%)	<0.05
>6 months	208	67 (32.2%)	141 (57.8%)	
Species				
Cats	270	97 (35.9%)	173 (64.1%)	>0.05
Dogs	123	49 (39.8%)	74 (60.2%)	
Seasons				
Spring	121	59 (48.8%)	62 (51.2%)	<0.05
Summer	101	32 (31.7%)	69 (68.3%)	
Autumn	43	7 (16.3%)	36 (83.7%)	
Winter	128	48 (37.5%)	80 (62.5%)	

**Table 2:** The coronavirus groups, genotypes and recombination event between symptomatic and asymptomatic animals.

	Symptomatic animals (*n* = 67)	Asymptomatic animals (*n* = 79)
FCoV	32 (47.8)	35 (44.3)
FCoV-I	11 (16.4)	11 (13.9)
CCoV	21 (31.3)	13 (16.5)
CCoV-I	4 (6.0)	0 (0.0)
CCoV-IIa	7 (10.4)	6 (7.6)
CCoV-IIb	0 (0.0)	1 (1.3)^*∗*^
Recombination event	—	C21041821-2 strain

^*∗*^This animal is infected with both CCoV-IIa and CCoV-IIb.

## Data Availability

All data of this study are available from the corresponding author upon request.

## Supplementary Materials

Supplementary Materials Table S1. Primers for detection and genotyping of CoV. Table S2. PCR procedure for the complete *S* gene amplification. Table S3. Primers for the complete *S* gene amplification. Table S4. Different clinical characteristics for CoV-positive cases. Table S5. The homologies analysis of the complete *S* genes in this study.
