# Supplementary material for: Epidemiology and Molecular Characterizations of Coronavirus from Companion Animals Living in Chengdu, Southwest China
Source: Transbound Emerg Dis. 2023 Mar 30;2023:5056492. doi: 10.1155/2023/5056492 (PMC12017059; doi:10.1155/2023/5056492)
Supplement: Supplementary Materials — Table S1. Primers for detection and genotyping of CoV. Table S2. PCR procedure for the complete S gene amplification. Table S3. Primers for the complete S gene amplification. Table S4. Different clinical characteristics for CoV-positive cases. Table S5. The homologies analysis of the complete S genes in this study. [file 5056492.f1.docx]

**Supplementary Materials**

Table S1. Primers for detection and genotyping of CoV

| Target gene | Primer name | Sequence (5′~3′) | length (bp) | Reference |
| --- | --- | --- | --- | --- |
| RdRp (detection) | pan-CoV-R | TGTTGIGARCARAAYTCATGIGG | 670~673 | Xiu et al. |
|  | pan-CoV-outF | CCAARTTYTAYGGHGGITGG |  |  |
|  | pan-CoV-R | TGTTGIGARCARAAYTCATGIGG | 599~602 |  |
|  | pan-CoV-inF | GGTTGGGAYTAYCCHAARTGTGA |  |  |
| S (typing) | FCoV-Ⅰ-nIffles | CCTAGAAAGCCTCAGATGAGTG | 360 | Addie et al. |
|  | FCoV-Ⅰ-nIubs | CCAAGGCCATTTTACATA |  |  |
|  | FCoV-Ⅱ-nIcfs | CAGACCAAACTGGACTGTAC | 218 |  |
|  | FCoV-Ⅱ-nIubs | CCAAGGCCATTTTACATA |  |  |
|  | CECoV-Ⅰ-EL1F | CAAGTTGACCGTCTTATTACTGGTAG | 346 | Pratelli et al. |
|  | CECoV-Ⅰ-EL1R | TCATATACGTACCATTATAGCTGAAGA |  |  |
|  | CECoV-Ⅱa-S5 | TGCATTTGTGTCTCAGACTT | 694 |  |
|  | CECoV-Ⅱa-S6 | CCAAGGCCATTTTACATAAG |  |  |
|  | CECoV-Ⅱb-CEPol-1 | TCTACAATTATGGCTCTATCAC | 370 | Erles et al. |
|  | CECoV-Ⅱb-TGSP-2 | TAATCACCTAAMACCACATCTG |  |  |

Table S2. PCR procedure for the complete S gene amplification

| Procedure | Temperature(℃) | Time | Cycle |
| --- | --- | --- | --- |
| Pre-denaturation | 94 | 5 min | 1 |
| Denaturation | 94 | 30 s | 35 |
| Annealing | Optimum annealing temperature (table S3) | 30 s |  |
| Extension | 72 | 1 min 30 s |  |

Table S3. Primers for the complete S gene amplification

| Primer name | Sequence (5′~3′) | length (bp) | Annealing temperature(℃) |
| --- | --- | --- | --- |
| FCoV/CECoV-Ⅰ-S1F | CTAAGGAAGGGTAAAATACTC | 1645 | 50.3 |
| FCoV/CECoV-Ⅰ-S1R | GCAGAATAAAAYCCATCTGGTA |  |  |
| FCoV/CECoV-Ⅰ-S2F | TATACAGATGTAATGGTGGATGT | 1610 | 53.7 |
| FCoV/CECoV-Ⅰ-S2R | CCATTRTAATATTGGGCACAAACTA |  |  |
| FCoV/CECoV-Ⅰ-S3F | ﻿TTGGCACTGTKGATGAWGA | 1386 | 53.7 |
| FCoV/CECoV-Ⅰ-S3R | ﻿TTTCAAATTGTCTTCTACTACA |  |  |
| FCoV/CECoV-Ⅱ-S1F | GGTTGTTGGATTACTAAGGAA | 1783 | 55.9 |
| FCoV/CECoV-Ⅱ-S1R | ATTAGCAGTAAGTTGAGAACA |  |  |
| FCoV/CECoV-Ⅱ-S2F | TGGTTACAATTTCTTTAGCAC | 1713 | 57.9 |
| FCoV/CECoV-Ⅱ-S2R | ACCATGATGCCATTGTAATAT |  |  |
| FCoV/CECoV-Ⅱ-S3F | GATCCTATTTACAAAGAATGG | 1565 | 50.3 |
| FCoV/CECoV-Ⅱ-S3R | ACATGCACTTTTTCAATTGGTTC |  |  |
| CRCoV-S1F | TGGGTGTTGCGGTCATAATTAT | 1051 | 53.7 |
| CRCoV-S1R | TGCAATTGGCTGAACAGTGTAAC |  |  |
| CRCoV-S2F | ATGCTGTTGATTGTAAGAGTGA | 1088 | 53.5 |
| CRCoV-S2R | AATACCTTGGCCTGTAATAC |  |  |
| CRCoV-S3F | ATAGTGGTACTACTTGTTCTACTG | 1207 | 55.9 |
| CRCoV-S3R | TGACTTAGCACATCCATGT |  |  |
| CRCoV-S4F | ﻿GTTGCCTCCACTGCTCTCAGA | 1309 | 61.3 |
| CRCoV-S4R | TGCTCGACCTCAATGGGTCT |  |  |

Table S4. Different clinical characteristics for CoV-positive cases.

| **Clinical charateristics** | **CoV-positive samples** | **FCov-positive samples** | **CCoV-positive samples** |
| --- | --- | --- | --- |
|  | (n=67) | (n=32) | (n=21) |
| Intestinal symptoms |  |  |  |
| Diarrhea | 28 (41.8) | 14 (43.8) | 13 (61.9) |
| Emesis | 24 (35.8) | 10 (31.2) | 9 (42.9) |
| Hematochezia | 12 (17.9) | 6 (18.8) | 3 (14.3) |
| Respiratory symptoms |  |  |  |
| Cough | 9 (13.4) | 5 (15.6) | 3 (14.3) |
| Runny nose | 6 (9.0) | 1 (3.1) | 3 (14.3) |
| Sneeze | 4 (6.0) | 1 (3.1) | 1 (4.8) |
| Fever (>37.5℃) | 4 (6.0) | 3 (9.4) | 1 (4.8) |

Table S5. The homologies analysis of the complete *S* genes in this study

| **strain** | **number** | **homology（%）** | | | |
| --- | --- | --- | --- | --- | --- |
|  |  | **nucleotide (nt)** | **amino acid (aa)** | | |
|  |  | **S** | **S** | **S1** | **S2** |
| FCoV | 2 | 87.10 | 90.39 | 85.69 | 94.72 |
| CECoV | 5 | 87.51~98.69 | 83.81~99.17 | 67.74~98.96 | 98.63～99.83 |
| CRCoV | 1 | / | / | / | / |
| Total | 8 | 52.79～98.69 | 27.08~99.17 | 15.25~98.96 | 35.90～99.83 |
